# Effects of graded phosphate deficiency and vitamin D intervention on growth, bone metabolism, and mineralization in a rat model of neonatal-onset metabolic bone disease

**DOI:** 10.1093/jbmrpl/ziag007

**Published:** 2026-01-16

**Authors:** Shanshan Wu, Huifeng Zhang

**Affiliations:** Department of Neonatology, Hebei Key Laboratory of Early Life Health Promotion (SZX202419), The Second Hospital of Hebei Medical University, Hebei Medical University, Shijiazhuang, 050000, Hebei Province, China; Department of Pediatrics, Hebei Key Laboratory of Early Life Health Promotion (SZX202419), The Second Hospital of Hebei Medical University, Hebei Medical University, Shijiazhuang, 050000, Hebei Province, China

**Keywords:** phosphate, vitamin D, growth, bone metabolism, bone mineralization, bone disease, growth plate, FGF23

## Abstract

To investigate the dose-dependent effects of neonatal-onset phosphate deficiency on bone growth and mineralization and assess whether supraphysiological vitamin D_3_ or calcitriol can rescue skeletal defects. Newborn Sprague-Dawley rats were randomly assigned to 7 diets: phosphate-free (0P), low phosphate (1/2P), normal phosphate (NP), calcium-free (0Ca), phosphate/vitamin D-free (0P/D), and 0P/D supplemented with either supraphysiological vitamin D_3_ (0P/D + D_3_) or calcitriol (0P/D + calcitriol). Longitudinal radiographic assessments were performed before euthanasia at 6-8 wk. Serum analyses measured phosphate (sP), calcium (sCa), phosphotropic hormones, and bone turnover markers. Tibial growth plates were examined by H&E staining, micro-CT, and histomorphometry. The 0P group developed severe hypophosphatemia, rickets-like growth plate widening, osteopenia, and growth retardation. The 1/2P group showed similar hypophosphatemia but no growth impairment and non-significant reductions in bone mass. The 0Ca group exhibited hypocalcemia, secondary hyperparathyroidism, and high bone resorption, yet maintained normal growth and intermediate mineralization. Vitamin D interventions normalized sP but worsened bone loss and growth impairment compared to the 0P group. Biochemically, sP correlated positively with bone formation markers and negatively with fibroblast growth factor-23 (FGF23); vitamin D showed dual effects on bone turnover. Phosphate sufficiency during the early postnatal period is critical for bone mineralization and growth in neonatal rats. Isolated calcium deficiency caused a distinct osteomalacic phenotype with preserved growth. Supraphysiological-dose vitamin D metabolites corrected hypophosphatemia but failed to rescue—and may have exacerbated—skeletal defects, cautioning against vitamin D monotherapy without concurrent phosphate supplementation.

## Graphical Abstract

**Graphical Abstract:**
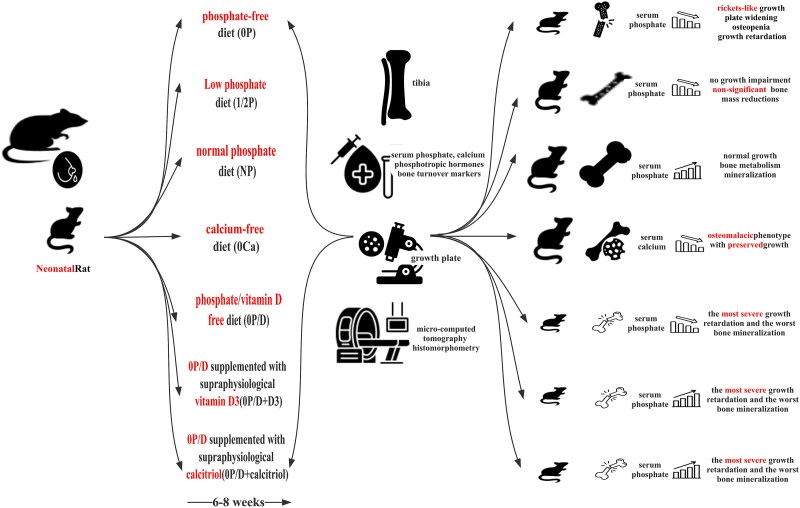

## Introduction

Metabolic bone disease of prematurity (MBDP), affecting 16%-40% of very low birth weight infants, is characterized by impaired bone mineralization and reduced bone mass.^1–3^ This condition leads to rickets-like changes, pathological fractures, and long-term growth impairment, with phosphate deficiency being a major pathogenic factor.^4–6^ The essential role of phosphate in skeletal development is demonstrated by hydroxyapatite, which accounts for approximately 85% of the body’s inorganic phosphate content and forms the principal mineral component of bone matrix. Beyond its structural function in bone, phosphate also participates in numerous physiological processes, including energy metabolism, cell signaling, and acid-base balance.^7^ Critically, phosphate is indispensable for the process of endochondral ossification, which drives longitudinal growth at the growth plate. Longitudinal bone growth occurs at the growth plate, where chondrocytes progress through resting, proliferative, and hypertrophic zones (HZs) before undergoing ossification and being replaced by bone. This highly coordinated process involves columnar proliferation of chondrocytes, vascular invasion, and subsequent replacement by osteoblast-deposited mineralized matrix.^8^^,^^9^

Despite this understanding, key questions remain regarding optimal phosphate intake levels and serum phosphate (sP) thresholds for maintaining neonatal bone mineralization.^10^^,^^11^ Clinical studies are limited by ethical constraints on manipulating infant phosphate intake and the inability to monitor growth plate histology. While animal studies have advanced calcium metabolism research, phosphate homeostasis has been relatively neglected, with most models examining calcium-phosphate ratios in adult rodents or genetic hypophosphatemia.^12^^,^^13^ A critical gap therefore exists in our understanding of phosphate-specific deficiency during early development. To address this, we developed a neonatal rat model that permits controlled dietary phosphate manipulation alongside direct growth plate assessment—a methodological advantage over clinical studies.

In this study, newborn Sprague-Dawley rats were randomized to 7 dietary groups: phosphate-free (0P), low phosphate (1/2P), normal phosphate (NP), calcium-free (0Ca), phosphate/vitamin D-free (0P/D), and 2 0P/D subgroups receiving either supraphysiological vitamin D_3_ (0P/D + D_3_) or calcitriol (0P/D + calcitriol). Lactating dams were maintained on experimental diets to induce nutrient-specific deficiencies through milk transmission.^14^ Pups were weaned onto assigned diets at 3 wk and evaluated at 6-8 wk through radiography, serum analyses, tibial growth plate histology, and micro-CT histomorphometry. This systematic approach was designed to comprehensively evaluate the effects of graded phosphate deficiency and vitamin D intervention on bone development and to determine whether vitamin D can rescue skeletal defects in the context of phosphate deprivation.

## Materials and methods

### Ethical approval and animal care

This study was approved by the Research Ethics Committee of the Second Hospital of Hebei Medical University (No. 2023-AE-120) and adhered to the Guide for the Care and Use of Laboratory Animals (eighth edition, NRC 2011). Seven pregnant Sprague-Dawley rats were obtained from China Xinbainuo Biological Technology Co., Ltd. After delivery, newborn pups from at least 3 litters were housed under controlled conditions (22 °C, 12-h light/dark cycle) with ad libitum access to food and water. Euthanasia was performed under sodium pentobarbital anesthesia (100 mg/kg, i.p.) followed by cervical dislocation, in accordance with AVMA Guidelines.

### Experimental design and randomization

Pregnant dams were randomly assigned to the 7 dietary groups using a computer-generated random number sequence. Within each litter, pups were then randomly distributed to the experimental groups to minimize litter effects. The groups were fed AIN-93G-based purified diets (ShuLaibao Biotechnology) as follows: (1) phosphate-free amino acid-based diet (0P), (2) low phosphate (0.15%) amino acid-based diet (1/2P), (3) normal phosphate diet (NP), (4) calcium-free casein-based diet (0Ca), (5) phosphate-free/vitamin D-deficient diet (0P/D), (6) 0P/D supplemented with vitamin D_3_ (200 IU/d; Double Whale Pharma), and (7) 0P/D with calcitriol (60 ng/d; Rocaltrol). During lactation, pups received exclusive maternal nutrition, constituting the neonatal-onset phase of the dietary interventions. At weaning (postnatal day 21), 8 pups per group (4 males and 4 females) continued their assigned diets until sacrifice at 6-8 wk. Due to the nature of the dietary interventions, blinding during animal handling was not feasible. However, investigators were rigorously blinded during all endpoint assessments, including serum biomarker analysis, histological evaluation, and micro-CT image analysis and quantification. All samples were coded prior to analysis to prevent bias.

### Animal welfare and adverse events

No unexpected adverse events or infections occurred. The observed growth retardation in deficiency groups was an expected outcome of the dietary intervention. All animals were monitored twice daily for welfare. The protocol did not require modification.

### Sample analyses, bone histology, and histomorphometry

At endpoint, animals underwent in vivo X-ray imaging followed by cardiac blood collection. Serum was separated by centrifugation and stored at −80 °C for subsequent analyses. Electrolytes [sP, serum calcium (sCa)] were measured using an automated analyzer, while bone turnover markers and phosphotropic hormones were quantified using commercial enzyme-linked immunosorbent assay (ELISA) kits from Jianglai Bio. Bone turnover markers included bone-specific alkaline phosphatase (BALP, Cat# JL15752), procollagen type I C-terminal propeptide (PICP, Cat# JL15704), osteocalcin (Cat# JL21019), tartrate-resistant acid phosphatase (TRACP, Cat# JL21113), and C-terminal telopeptide of type I collagen (CTX-I, Cat# JL15706). Phosphotropic hormones comprised 25OHD (Cat# JL20733), 1,25(OH)₂D (Cat# JL20725), fibroblast growth factor-23 (FGF23, Cat# JL14035), intact PTH (iPTH, Cat# JL21082), and calcitonin (Cat# JL13263).

Left tibiae were harvested for histology, fixed in 4% neutral buffered formalin, decalcified in 14% EDTA (pH 7.4), and processed for H&E staining. Growth plate morphology was assessed by measuring proliferative (PZ) and hypertrophic (HZ) zone widths using OLYMPUS cellSens Dimension imaging software (Olympus Corporation), with HZ% calculated as HZ/(PZ + HZ).^8^^,^^15^

Tibiae from 6- to 8-wk-old rats were scanned using a SkyScan 1176 micro-CT system (Bruker) at 18 μm isotropic resolution. To enable an unbiased comparison of trabecular bone parameters across groups with differing tibial lengths, a standardized region of interest (ROI) was defined within the secondary spongiosa of each specimen. This ROI was positioned starting at a fixed anatomical point (0.7 mm distal to the growth plate-metaphyseal junction^14^) and extended distally for a distance equivalent to 5% of the total tibial length. This length-normalized approach ensures that the analyzed metaphyseal volume is anatomically comparable relative to overall bone size, which is critical for the valid comparison of volumetric measures, such as BMD and bone volume fraction (BV/TV). Following acquisition, images were reconstructed using NRecon software (Bruker, version 1.7.0.4) with consistent grayscale thresholds (51-255), then analyzed in CTAn software (Bruker, version 1.17.7.2+) to quantify BMD, trabecular microarchitecture, and geometric properties in accordance with the standard guidelines from the American Society for Bone and Mineral Research,^16^ ensuring exclusion of primary spongiosa from measurement.^14^^,^^17^

### Sample size and statistical analyses

Based on the pilot data, which identified sP as the key outcome, a sample size calculation was performed. With an expected mean difference of 0.58 mmol/L, a SD of 0.4, a power of 80%, and an α-level of .05, the analysis indicated that a minimum of 6 animals per group was required to detect a statistically significant effect. To account for potential attrition and to ensure balanced groups with equal numbers of males and females (*n* = 4 each), the sample size was increased to 8 animals per group. Data distribution was assessed using the Shapiro–Wilk test, and homogeneity of variances was verified using Levene’s test. Normally distributed data are presented as mean ± SD, while non-normally distributed data are presented as median with interquartile range. For intergroup comparisons: if data satisfied both normality and homogeneity of variances, one-way analysis of variance (ANOVA) was employed, followed by Fisher’s least significant difference test for post-hoc pairwise comparisons if the ANOVA was significant. If the assumptions of normality or homogeneity of variances were violated, the non-parametric Kruskal–Wallis H test was used, followed by Bonferroni-corrected Mann–Whitney U tests for post-hoc pairwise comparisons if the Kruskal–Wallis test was significant. Correlations were assessed using Pearson’s or Spearman’s tests as appropriate. Male and female rats were equally distributed across all groups to control for potential sex-related variation. The absence of a significant diet-by-sex interaction was confirmed via 2-way ANOVA for all primary outcomes (including weight at termination, tibia length, BMD, bone volume, sCa, sP, 25OHD, and HZ widths; all *p* > .05). Consequently, data from both sexes were combined for all group comparisons presented. All analyses were performed with SPSS 27.0.1 (IBM).

## Results

### Phenotypic observations (growth parameters and imaging manifestations)

**Figure 1:**
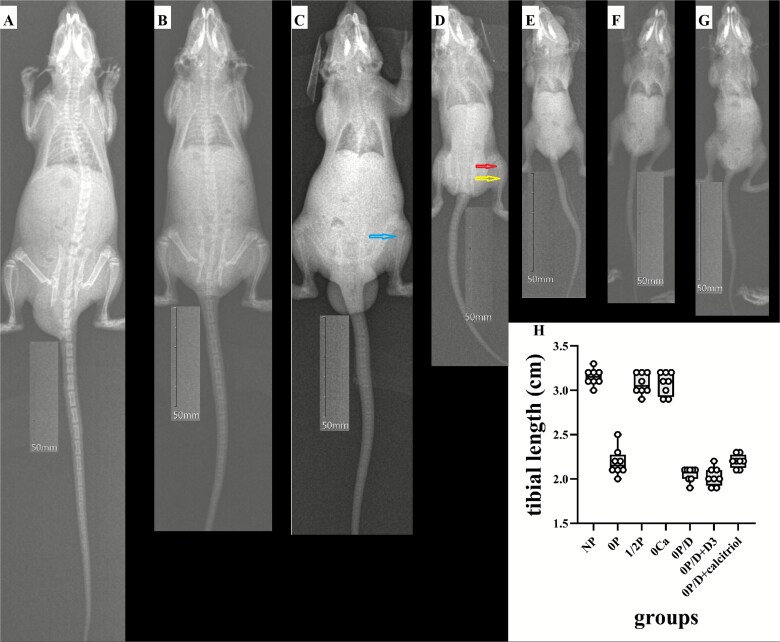
Representative in vivo radiographs and lengths of tibiae from neonatal rats under dietary interventions. Radiographic assessment reveals distinct skeletal phenotypes induced by specific nutrient deficiencies. (A) NP group: normal tibial architecture with sharp cortical margins. (B) 1/2P group: slightly fainter margins without significant shortening. (C) 0Ca group: features of subperiosteal resorption (blue arrow) with preserved bone length. (D) 0P group: severe rachitic changes, including blurred margins, decreased radiopacity (yellow arrow), characteristic “brush-like” growth plates (red arrow), and a significant 30% reduction in bone length (*p* < .001). (E-G) Severely hypomineralized tibiae from the (E) 0P/D, (F) 0P/D + D_3_, and (G) 0P/D + calcitriol groups, which required high exposure indices for adequate visualization and exhibited substantial length reductions. Scale bar, 50 mm (applicable to all panels). (H) Data are presented as box plots showing the median (center line), the 25th and 75th percentiles (bounds of box), and the minimum and maximum values (whiskers). Individual data points (*n* = 8 per group) are overlaid. Significant reduction in tibial length was observed in the 0P, 0P/D, 0P/D + D_3_, and 0P/D + calcitriol groups compared to the NP group. ^**^p < .001 vs NP. Abbreviations: 0P, phosphate-free; 1/2P, low phosphate; NP, normal phosphate; 0Ca, calcium-free; 0P/D, phosphate/vitamin D-free; 0P/D + D_3_, 0P/D supplemented with supraphysiological vitamin D_3_; 0P/D + calcitriol, 0P/D supplemented with supraphysiological calcitriol.

All neonatal rat groups demonstrated comparable genetic backgrounds and similar birth weights at baseline (*p* > .05). Growth analysis revealed that tibial length measurements were consistently correlated with body weight gain throughout the study (Table 1). At termination, the 0P, 0P/D, and vitamin D-treated groups (0P/D + D_3_ and 0P/D + calcitriol) exhibited significantly impaired growth, showing 65%-85% reductions in weight gain and 30% shorter tibial lengths compared to NP controls (*p* < .001). The 1/2P and 0Ca groups displayed modest 8%-10% decreases that did not reach statistical significance. Radiographic assessment under standard exposure conditions (Exposure Index = 141) revealed distinct morphological differences (Figure 1). Normal phosphate controls maintained normal tibial architecture with sharp cortical margins, while the 0P group presented blurred margins (yellow arrow), decreased radiopacity, and a characteristic “brush-like” growth plate (red arrow) with an accompanying 30% reduction in tibial length (*p* < .001). The 1/2P group displayed slightly fainter margins without significant shortening, and the 0Ca group exhibited subperiosteal resorption (blue arrow) but preserved bone length. Notably, the 0P/D and vitamin D-treated groups required extreme exposure indices for tibial visualization, which also revealed approximately 30% length reductions compared to NP. These radiographic observations of impaired mineralization and structural deterioration were unequivocally confirmed and presented in greater detail by subsequent high-resolution micro-CT imaging (see “Micro-CT analysis” section below).

**Table 1:** Growth parameters in neonatal rats fed phosphate/calcium-deficient diets with vitamin D interventions.

	**BW (g)**	**BW_Term(g)**	**WG (g)**	**TL (cm)**
**NP**	6.8 ± 0.2	111.8 ± 9.2	105.1 ± 9.3	3.2 ± 0.1
**0P**	6.8 ± 0.2	41.7 ± 4.5^*^	34.9 ± 4.4^*^	2.2 ± 0.2^**^
**1/2P**	6.7 ± 0.2	100.2 ± 6.8	93.5 ± 6.9	3.1 ± 0.1
**0Ca**	6.9 ± 0.2	103.0 ± 9.0	96.2 ± 9.2	3.1 ± 0.1
**0P/D**	6.8 ± 0.3	22.1 ± 2.3^**^	15.3 ± 2.6^**^	2.1 ± 0.1^**^
**0P/D + D**_**3**_	7.0 ± 0.3	19.8 ± 1.3^**^	12.9 ± 1.2^**^	2.0 ± 0.1^**^
**0P/D + calcitriol**	6.8 ± 0.3	25.4 ± 2.1^**^	18.6 ± 2.1^**^	2.2 ± 0.1^**^
***p*-Value**	.511 (F = 0.888)	<.001 (H = 50.198)	<.001 (H = 50.142)	<.001 (F = 185.925)

^*^ *p* < .05, ^**^p < .001 vs NP group. Abbreviations: 0P, phosphate-free; 1/2P, low phosphate; NP, normal phosphate; 0Ca, calcium-free; 0P/D, phosphate/vitamin D-free; 0P/D + D_3_, 0P/D supplemented with supraphysiological vitamin D_3_; 0P/D + calcitriol, 0P/D supplemented with supraphysiological calcitriol; BW, birth weight; BW_Term, weight at termination; WG, weight gain; TL, tibial length.

### Mineral metabolism and bone turnover markers

**Figure 2:**
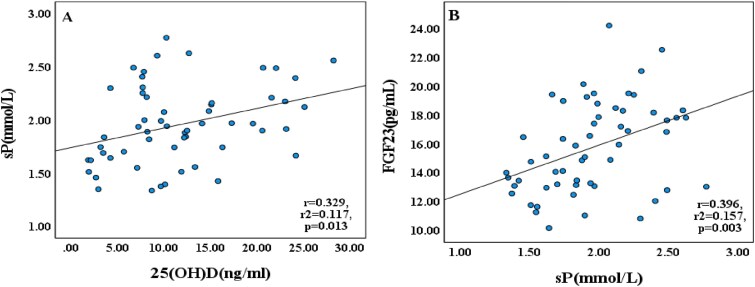
Correlations between serum phosphate and phosphotropic hormones. Linear regression analyses of serum parameters in neonatal rats under dietary interventions: (A) 25OHD vs sP and (B) sP vs FGF23. Data from all experimental groups were pooled for analysis (*n* = 56). Solid lines indicate the line of best fit. Abbreviations: 25OHD, serum 25-hydroxyvitamin D; sP, serum phosphate; FGF23, serum fibroblast growth factor-23.

Serum analyses (Table 2) demonstrated severe hypophosphatemia in the 0P, 1/2P, and 0P/D groups (*p* < .001), which was normalized by vitamin D interventions. The 0Ca group developed marked hypocalcemia without hyperphosphatemia. Hormonal profiling revealed the 0P/D group exhibited the lowest circulating levels of both 25OHD and among all groups (*p* < .001), whereas the 0Ca group demonstrated marked elevations in these metabolites accompanied by substantially increased iPTH levels (*p* < .001). Serum FGF23 levels peaked in the 0Ca and 0P/D + calcitriol groups (*p* < .001). Correlation analyses revealed significant associations between parameters (Figure 2): sP positively correlated with 25OHD (*r* = 0.329, *p* = .013) and FGF23 (*r* = 0.396, *p* = .003). Additionally, several key relationships within the broader mineral homeostasis network were observed, including inverse correlations between sCa and iPTH, and positive correlations among vitamin D metabolites and phosphotropic hormones (Figure S1).

**Table 2:** Mineral homeostasis and bone remodeling responses to phosphate/calcium modulation with vitamin D supplementation.

	**NP**	**0P**	**1/2P**	**0Ca**	**0P/D**	**0P/D + D**_**3**_	**0P/D + calcitriol**	***p*-Value**
**Ca (mmol/L)**	2.43 ± 0.25	2.54 ± 0.08	2.50 ± 0.05	1.73 ± 0.12^**^	2.36 ± 0.19	2.67 ± 0.51	2.41 ± 0.26	<.001 (H = 24.245)
**P (mmol/L)**	2.29 ± 0.36	1.60 ± 0.23^**^	1.75 ± 0.23^**^	2.18 ± 0.30	1.70 ± 0.39^**^	2.01 ± 0.21	2.16 ± 0.20	<.001 (F = 7.252)
**25OHD (ng/mL)**	11.22 (6.97, 14.49)	7.91 (3.74, 9.68)	12.84 (11.98, 14.65)	23.09 (20.06, 24.88)^*^	2.83 (1.99, 5.07)^*^	14.89 (8.89, 22.41)	8.03 (7.72, 13.41)	<.001 (H = 34.785)
**1,25(OH)**_**2**_ **D (pg/mL)**	28.65 (18.52, 42.46)	17.88 (10.09, 24.17)	30.25 (27.72, 37.50)	58.32 (48.87, 60.07)^*^	10.39 (4.82, 14.96)^*^	27.87 (23.37, 36.74)	28.32 (20.89, 49.90)	<.001 (H = 34.008)
**iPTH (pg/mL)**	10.77 (6.33, 13.85)	7.54 (3.75, 9.02)	14.09 (13.17, 14.89)	20.08 (18.51, 25.34)^*^	8.13 (6.99, 10.28)	12.42 (8.62, 19.04)	8.17 (7.47, 9.19)	<.001 (H = 27.988)
**FGF23 (pg/mL)**	14.32 ± 2.47	13.37 ± 2.56	14.31 ± 1.65	18.90 ± 2.36^**^	15.05 ± 2.64	15.61 ± 3.99	18.65 ± 1.58^**^	<.001 (F = 5.773)
**BALP (U/L)**	19.52 ± 2.79	19.49 ± 4.25	20.95 ± 5.24	25.91 ± 3.54^*^	20.86 ± 5.05	30.45 ± 4.29^**^	29.31 ± 3.16^**^	<.001 (F = 10.338)
**PICP (ng/mL)**	25.09 ± 2.88	25.10 ± 2.06	23.58 ± 1.66	22.85 ± 2.69	21.21 ± 2.84 ^*^	23.36 ± 2.77	22.88 ± 1.71	.034 (F = 2.514)
**osteocalcin (ng/mL)**	0.18 ± 0.03	0.18 ± 0.03	0.20 ± 0.04	0.26 ± 0.04^**^	0.23 ± 0.05^*^	0.26 ± 0.02^**^	0.24 ± 0.02^**^	<.001 (F = 8.051)
**TRACP (ng/mL)**	8.02 ± 1.52	7.09 ± 0.74	6.91 ± 1.32	10.89 ± 1.26^**^	6.84 ± 1.45	7.96 ± 1.07	6.81 ± 1.65	<.001 (F = 9.844)
**CTX-I (ng/mL)**	0.29 ± 0.06	0.26 ± 0.03	0.24 ± 0.05	0.40 ± 0.05^*^	0.25 ± 0.07	0.20 ± 0.11	0.24 ± 0.06	.001 (H = 23.532)
**calcitonin (pg/mL)**	411.07 ± 34.86	411.93 ± 18.86	407.41±21.79	338.68±19.42^**^	408.52±15.98^*^	499.33 ± 89.13^**^	467.58 ± 78.59^*^	<.001 (H = 26.037)

^*^ *p* < .05, ^**^p ≤ .001 vs NP group. Abbreviations: 0P, phosphate-free; 1/2P, low phosphate; NP, normal phosphate; 0Ca, calcium-free; 0P/D, phosphate/vitamin D-free; 0P/D + D_3_, 0P/D supplemented with supraphysiological vitamin D_3_; 0P/D + calcitriol, 0P/D supplemented with supraphysiological calcitriol; Ca, serum calcium; P, serum phosphate; 25OHD, serum 25-hydroxyvitamin D; 1,25(OH)_2_D, serum 1,25-dihydroxyvitamin D; iPTH, serum intact PTH; FGF23, serum fibroblast growth factor-23; BALP, serum bone-specific alkaline phosphatase; PICP, serum procollagen type I C-terminal propeptide; TRACP, serum tartrate-resistant acid phosphatase; CTX-I, serum C-terminal telopeptide of type I collagen.

**Figure 3:**
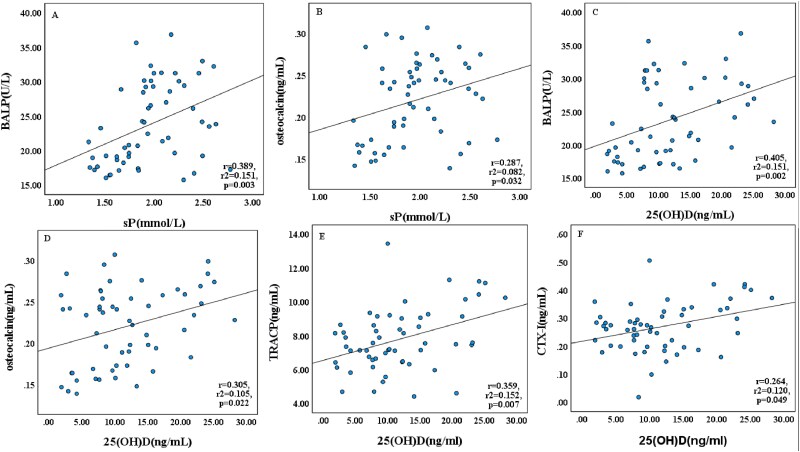
Correlations between serum phosphate, 25OHD, and bone turnover markers. Linear regression analyses of serum phosphate, 25OHD, and bone turnover markers in neonatal rats under dietary interventions: (A) sP vs BALP, (B) sP vs osteocalcin, (C) 25OHD vs BALP, (D) 25OHD vs osteocalcin, (E) 25OHD vs TRACP, and (F) 25OHD vs CTX-I. Data from all experimental groups were pooled for analysis (*n* = 56). Solid lines indicate the line of best fit. Abbreviations: sP, serum phosphate; BALP, bone-specific alkaline phosphatase; 25OHD, serum 25-hydroxyvitamin D; TRACP, tartrate-resistant acid phosphatase; CTX-I, C-terminal telopeptide of type I collagen.

**Figure 4:**
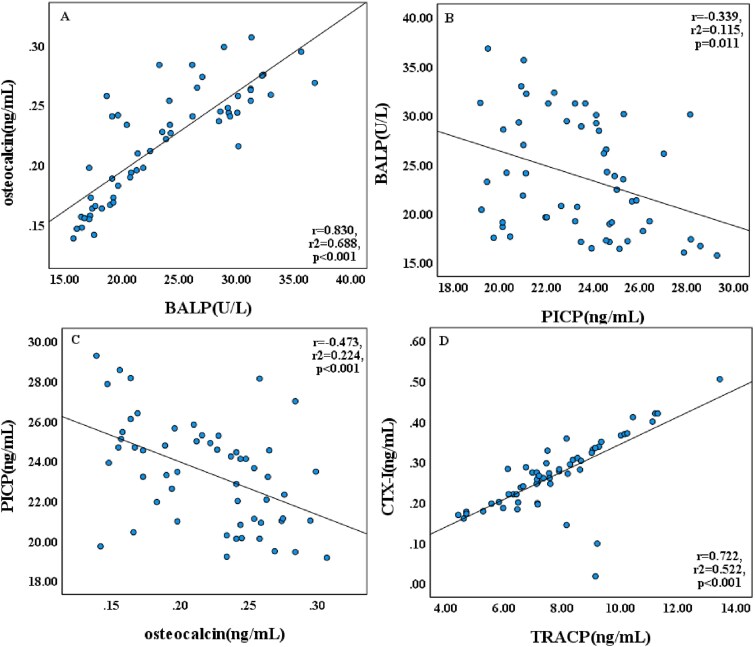
Correlations between serum bone turnover markers. Linear regression analyses of serum bone turnover markers in neonatal rats under dietary interventions: (A) BALP vs osteocalcin, (B) PICP vs BALP, (C) osteocalcin vs PICP, and (D) TRACP vs CTX-I. Data from all experimental groups were pooled for analysis (*n* = 56). Solid lines indicate the line of best fit. Abbreviations: BALP, bone-specific alkaline phosphatase; PICP, procollagen I C-terminal propeptide; TRACP, tartrate-resistant acid phosphatase; CTX-I, C-terminal telopeptide of type I collagen.

Bone turnover markers exhibited distinct patterns in relation to mineral and vitamin D status (Table 2 and Figure 3). Formation markers (BALP, osteocalcin) showed no significant differences between 0P, 1/2P, and NP groups (*p* > .05) but demonstrated 40%-55% increases in vitamin D-treated groups (*p* < .001). Positive correlations were observed between sP and BALP (*r* = 0.389, *p* = .003) as well as osteocalcin (*r* = 0.287, *p* = .032). Similarly, 25OHD levels correlated positively with both BALP (*r* = 0.405, *p* = .002) and osteocalcin (*r* = 0.305, *p* = .022). Resorption markers showed significant alterations in the 0Ca group, with TRACP and CTX-I levels markedly elevated by approximately 40% (*p* < .001) while calcitonin was significantly reduced by 17.6% (*p* < .001). Serum calcium showed a strong positive correlation with calcitonin (Figure S2). Positive associations were observed between 25OHD levels and both TRACP (*r* = 0.359, *p* = .007) and CTX-I (*r* = 0.264, *p* = .049). Interrelationships among bone turnover markers revealed consistent patterns (Table 2 and Figure 4). Formation markers BALP and osteocalcin showed strong positive correlation (*r* = 0.830, *p* < .001), while both markers inversely correlated with PICP (BALP: *r* = −0.339, *p* = .011; osteocalcin: *r* = −0.473, *p* < .001). Among resorption markers, TRACP and CTX-I were positively associated (*r* = 0.722, *p* < .001). Calcitonin levels demonstrated negative correlations with both TRACP and CTX-I (Figure S3).

### Bone microarchitecture analysis (histology and micro-CT)

**Figure 5:**
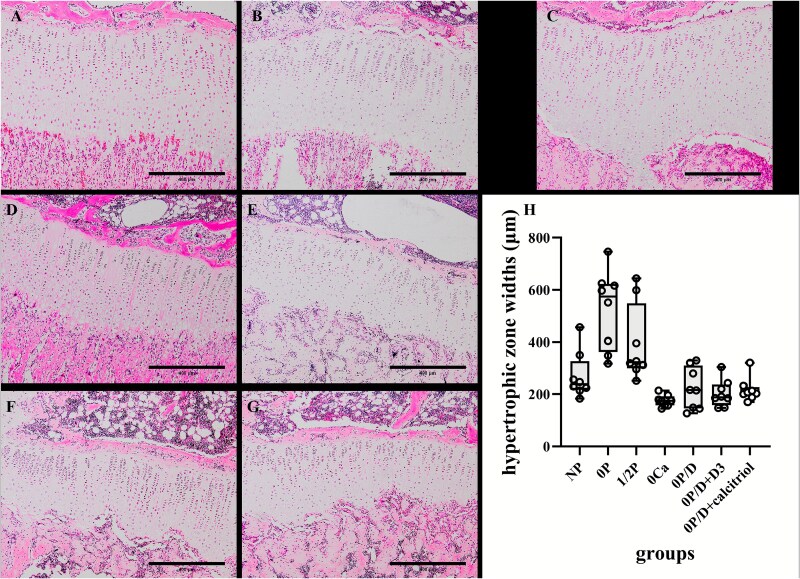
Comparative histological analysis of the proximal tibial growth plate. (A-G) Representative H&E-stained sections of the tibial growth plate from neonatal rats under dietary interventions. (A) NP group: normal growth plate architecture. (B) 1/2P group: modest, non-significant expansion. (C) 0P group: markedly expanded hypertrophic zone (HZ) and significantly increased total width. (E) 0P/D group: slight decrease in size. (D) 0Ca (calcium-free) group, (F) 0P/D + D_3_ group, and (G) 0P/D + calcitriol group: significantly reduced HZ and total growth plate widths. Scale bar, 400 μm (applicable to all panels). (H) Data are presented as box plots showing the median (center line), the 25th and 75th percentiles (bounds of box), and the minimum and maximum values (whiskers). Individual data points (*n* = 8 per group) are overlaid. The HZ width was significantly wider in the 0P group and significantly narrower in the 0Ca, 0P/D + D_3_, and 0P/D + calcitriol groups compared to the NP group. ^**^p < .001 vs NP. Abbreviations: NP, normal phosphate; 1/2P, low phosphate; 0P, phosphate-free; 0Ca, calcium-free; 0P/D, phosphate/vitamin D-free; 0P/D + D_3_, 0P/D supplemented with supraphysiological vitamin D_3_; 0P/D + calcitriol, 0P/D supplemented with supraphysiological calcitriol.

Comparative histological analysis of growth plates revealed significant morphological differences across experimental groups (Table 3 and Figure 5). The 0P group displayed markedly increased widths of both the HZ and PZ + HZ compared to NP controls (*p* < .001). While the 1/2P group exhibited modest, non-significant increases in these parameters, the 0P/D group showed slight but non-significant decreases. In contrast, the 0Ca group had significantly reduced HZ width vs NP (*p* < .05).

**Table 3:** Growth plate zonal dimensions following phosphate/calcium restriction and vitamin D rescue.

	**HZ (μm)**	**HZ + PZ (μm)**	**HZ% (%)**
**NP**	238.2 (216.4326.8)	442.6 (369.8549.3)	58.8 (50.7,61.2)
**0P**	574.4 (362.5622.9)^*^	831.9 (590.8882.0)^*^	67.1 (61.1,73.7)^*^
**1/2P**	322.8 (299.8548.0)	553.6 (475.6777.1)	63.0 (55.8,70.5)
**0Ca**	174.3 (157.0,191.6)^*^	342.2 (298.7404.4)	50.9 (48.4,52.6)
**0P/D**	215.5 (145.2310.5)	387.2 (251.5496.4)	57.3 (53.0,62.8)
**0P/D + D**_**3**_	187.6 (157.6237.1)	342.5 (297.8397.4)	59.7 (50.5,63.4)
**0P/D + calcitriol**	204.7 (190.7227.3)	332.6 (316.2363.7)	63.0 (59.0,64.3)
***p*-Value**	<.001 (H = 33.511)	<.001 (H = 33.848)	<.001 (H = 24.380)

^*^ *p* < .05 vs NP group. Abbreviations: 0P, phosphate-free; 1/2P, low phosphate; NP, normal phosphate; 0Ca, calcium-free; 0P/D, phosphate/vitamin D-free; 0P/D + D_3_, 0P/D supplemented with supraphysiological vitamin D_3_; 0P/D + calcitriol, 0P/D supplemented with supraphysiological calcitriol; HZ, hypertrophic zone; HZ + PZ, the combined width of the proliferative and hypertrophic zones; HZ%, the relative hypertrophic zone length.

**Figure 6:**
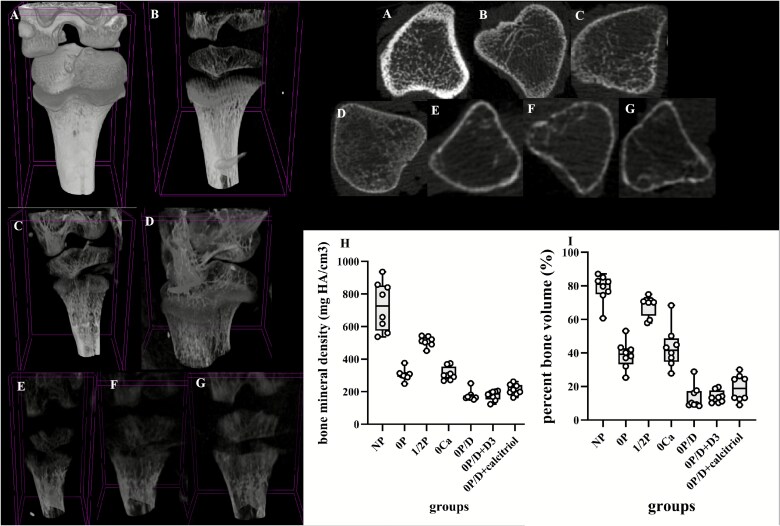
The micro-CT analysis of tibial bone changes in neonatal rats under various dietary interventions. Representative three-dimensional micro-CT reconstructions and standardized cross-sectional images of the proximal tibia from neonatal rats under dietary interventions. Images are qualitatively presented to visualize differences in bone density, mass, and architecture. (A) NP group: a dense, well-mineralized trabecular network, indicative of robust bone quality. (B) 1/2P group: mildly reduced density without severe structural loss. (C) 0P group: severe osteopenia, characterized by drastically reduced bone density, trabecular fragmentation, and loss of structural connectivity—hallmark features of rachitic deterioration. (D) 0Ca group: a distinct osteoporotic phenotype with thinned, sparse trabeculae but preserved overall structure. (E) 0P/D, (F) 0P/D + D_3_, and (G) 0P/D + calcitriol groups: more severe deterioration, showing profound hypomineralization and architectural disintegration. (H) Data are presented as box plots showing the median (center line), the 25th and 75th percentiles (bounds of box), and the minimum and maximum values (whiskers). Individual data points (*n* = 8 per group) are overlaid. Significant reductions in BMD were observed in the 0P, 0Ca, 0P/D, 0P/D + D_3_, and 0P/D + calcitriol groups compared to the NP group. ^**^p < .001 vs NP. (I) Data are presented as box plots showing the median (center line), the 25th and 75th percentiles (bounds of box), and the minimum and maximum values (whiskers). Individual data points (*n* = 8 per group) are overlaid. Significant reductions in BV/TV were observed in the 0P, 0Ca, 0P/D, 0P/D + D_3_, and 0P/D + calcitriol groups compared to the NP group. ^**^p < .001 vs NP. Abbreviations: micro-CT, micro-computed tomography; NP, normal phosphate; 1/2P, low phosphate; 0P, phosphate-free; 0Ca, calcium-free; 0P/D, phosphate/vitamin D-free; 0P/D + D_3_, 0P/D supplemented with supraphysiological vitamin D_3_; 0P/D + calcitriol, 0P/D supplemented with supraphysiological calcitriol; BV/TV, percent bone volume.

Micro-CT analysis of proximal tibial metaphyses revealed profound differences in trabecular bone architecture across groups (Table 4, Figure 6). The NP group demonstrated significantly superior bone mass and quality, with higher values for BMD, BV/TV, and trabecular thickness (Tb.Th) compared to all other groups (*p* < .001). In contrast, the phosphate-deficient groups (0P, 0P/D, and vitamin D-treated groups) exhibited marked reductions in these parameters (*p* < .001). The 1/2P group showed non-significant decreasing trends, while the 0Ca group had significantly lower values vs NP controls. Assessment of trabecular microstructure showed severe deterioration in the 0P/D, 0P/D + D_3_, and 0P/D + calcitriol groups. These groups displayed significantly lower trabecular number (Tb.N) and connectivity porosity (Pocl) and higher trabecular separation (Tb.Sp), trabecular pattern factor (Tb.Pf), and structure model index compared to controls. These changes reflect a transition from plate-like to rod-like trabeculae, characterized by fragmentation, perforation, and loss of connectivity, which collectively compromise mechanical strength. This interpretation is further supported by the increased degree of anisotropy (DA) observed in these groups. Both the 0P and 0Ca groups also showed significantly higher bone surface density (BS/TV) and Tb.Pf vs the NP group, indicating a shift toward thinner, more fragmented trabeculae and an increased osteoporosis risk. Representative 3D reconstructions and cross-sectional images visually confirmed these quantitative findings (Figure 6).

**Table 4:** Trabecular microarchitecture of the proximal tibial metaphysis by micro-CT.

	**NP**	**0P**	**1/2P**	**0Ca**	**0P/D**	**0P/D + D**_**3**_	**0P/D + calcitriol**	***p*-Value**
**BMD (mg HA/cm** ^**3**^**)**	727 (574, 853)	302 (291, 312)^*^	510 (493, 535)	300 (276, 354)^*^	165 (162, 178)^**^	187 (153, 200)^**^	205 (188, 241)^**^	<.001 (H = 49.814)
**BV/TV (%)**	81.07 (75.00, 84.49)	39.20 (33.28, 42.88)^*^	70.45 (62.22, 71.51)	41.64 (34.71, 48.82)^*^	9.78 (9.00, 17.27)^**^	13.41 (10.50, 17.59)^**^	18.76 (12.74, 25.75)^**^	<.001 (H = 48.274)
**BS/TV (1/mm)**	5.86 ± 0.51	7.60 ± 0.25^*^	6.85 ± 0.32	8.04 ± 0.67^*^	4.98 ± 0.45	3.26 ± 0.60^**^	3.75 ± 0.57^*^	<.001 (F = 13.617)
**Tb.Pf (1/mm)**	0.55 ± 0.09	3.99 ± 0.99	1.31 ± 0.26	5.03 ± 0.94^*^	14.48 ± 1.61^**^	18.87 ± 2.88^**^	11.63 (8.89, 14.61)^**^	<.001 (H = 44.962)
**SMI**	0.63 (0.35, 0.80)	1.11 (0.28, 1.73)	0.91 (0.25, 1.13)	1.93 (1.76, 2.04)^*^	2.21 (1.94, 2.43)^**^	2.28 (2.00, 2.84)^**^	2.17 (1.85, 2.58)^**^	<.001 (H = 39.294)
**Tb.Th (mm)**	0.39 (0.33, 0.42)	0.19 (0.17, 0.25)^*^	0.35 (0.30, 0.38)	0.17 (0.15, 0.19)^*^	0.10 (0.09, 0.15)^**^	0.12 (0.09, 0.15)^**^	0.12 (0.11, 0.15)^**^	<.001 (H = 44.116)
**Tb.N (1/mm)**	2.06 (1.98, 2.18)	1.86 (1.72, 2.01)	1.91 (1.76, 1.99)	2.03 (1.89, 2.72)	0.96 (0.89, 1.53)^**^	1.07 (0.93, 1.17)^**^	1.21 (0.96, 1.59)^**^	<.001 (H = 40.592)
**Tb.Sp (mm)**	0.23 (0.18, 0.27)	0.65 (0.34, 0.69)^*^	0.21 (0.18, 0.30)	0.28 (0.26, 0.32)	0.82 (0.70, 0.94)^**^	0.86 (0.65, 0.99)^**^	0.84 (0.64, 0.98)^**^	<.001 (H = 42.869)
**Pocl (%)**	0.60 (0.37, 0.91)	0.12 (0.10, 0.19)^*^	0.30 (0.24, 0.34)	0.20 (0.12, 0.31)	0.01 (0.00, 0.02)^**^	0.00 (0.00, 0.01)^**^	0.01 (0.00, 0.03)^**^	<.001 (H = 47.079)
**DA**	1.33 (1.30, 1.38)	1.65 (1.48, 1.86)	1.43 (1.40, 1.48)	2.00 (1.99, 2.00)^**^	2.80 (2.26, 2.95)^**^	2.63 (2.10, 2.99)^**^	2.12 (1.99, 2.24)^**^	<.001 (H = 42.416)

^*^ *p* < .05, ^**^p ≤ .001 vs NP group. Abbreviations: 0P, phosphate-free; 1/2P, low phosphate; NP, normal phosphate; 0Ca, calcium-free; 0P/D, phosphate/vitamin D-free; 0P/D + D_3_, 0P/D supplemented with supraphysiological vitamin D_3_; 0P/D + calcitriol, 0P/D supplemented with supraphysiological calcitriol; TV, tissue volume; BV, bone volume; BV/TV, percent bone volume; BS, bone surface; BS/TV, bone surface density; Tb.Pf, trabecular pattern factor; SMI, structure model index; Tb.Th, trabecular thickness; Tb.N, trabecular number; Tb.Sp, trabecular separation; Pocl, closed porosity (percent); DA, degree of anisotropy.

## Discussion

Our neonatal rat model successfully replicated the metabolic bone disease phenotype through neonatal-onset phosphate deprivation, demonstrating characteristic hypophosphatemia, growth plate widening, rickets, osteopenia, and growth retardation.^9^^,^^14^^,^^15^^,^^18^^,^^19^ Notably, while 50% phosphate restriction induced marked hypophosphatemia, only a subset of animals exhibited growth plate HZ widening. Radiographic and micro-CT imaging revealed reduced bone mass compared to NP controls, though histomorphometric analysis showed no statistically significant reductions in bone parameters or growth retardation. In contrast, calcium deprivation resulted in distinct skeletal changes, including growth plate narrowing and reductions in bone density and mass, manifesting as an osteoporosis-like phenotype with thinner, sparser trabeculae.^15^ Importantly, these calcium deficiency-induced abnormalities were less pronounced than those observed in complete phosphate deprivation. A particularly striking finding was that while supraphysiological dose vitamin D_3_ or calcitriol supplementation corrected hypophosphatemia, it failed to ameliorate skeletal abnormalities or growth retardation.^20–23^ These results establish phosphate during early life as the critical regulator of skeletal growth and mineralization in neonatal rodents, with deficiency exerting more detrimental effects than either calcium or vitamin D deficiency.^9^^,^^15^ These findings provide a mechanistic framework for improving MBDP prevention and clinical management.

The integrated mother-offspring axis in mineral homeostasis is a crucial consideration in interpreting our model. During the lactation period, neonatal rats received nutritional interventions exclusively through maternal milk, an established approach for studying developmental mineral deficiencies.^14^ While this design precludes direct measurement of milk composition, it accurately recapitulates the physiological scenario where the neonate is entirely dependent on nutrients processed by the mother. It is well-established that lactating dams can homeostatically regulate milk calcium, in part through increased skeletal resorption.^24^ The significant hypocalcemia nevertheless observed in our 0Ca group offspring demonstrates that this maternal buffering capacity was overwhelmed under severe dietary restriction, confirming the efficacy of our model in inducing a deficient state. This stands in stark contrast to the profound defects in phosphate-deficient groups, highlighting a greater vulnerability to phosphate restriction, likely because phosphate homeostasis lacks a similarly large, readily mobilizable skeletal reservoir. Thus, our findings not only delineate the skeletal consequences of specific nutrient deficiencies but also provide insights into the differential capacities of maternal homeostatic systems.

From a growth perspective, the NP group showed significantly greater gains in body weight and tibial length compared to phosphates-deprived groups. While 50% phosphate restriction had no significant effect on growth parameters, complete deprivation initiated neonatally led to marked growth retardation. Neither supraphysiological dose vitamin D_3_ nor calcitriol supplementation improved these growth impairments; rather, they exacerbated the deficits beyond those observed in the 0P group. In contrast, calcium deprivation did not significantly affect weight gain or longitudinal bone growth. These experimental findings align with clinical observations that MBDP is associated with persistent growth deficits,^4^^,^^5^ highlighting the importance of early phosphate supplementation for optimal growth outcomes. Clinical evidence confirms that early phosphate supplementation improves bone biomarkers, reduces MBDP incidence, and optimizes growth in preterm infants, underscoring the critical window for mineral intervention.^1^^,^^2^^,^^7^^,^^25^

Biochemically, when dietary manipulation was initiated neonatally, phosphate-deficient groups developed hypophosphatemia significantly, confirming that isolated phosphate deficiency disrupts mineral balance. Interestingly, sP levels did not show progressive decreases with further phosphate restriction (from 1/2P to 0P), suggesting compensatory skeletal phosphate mobilization to maintain circulating phosphate levels for essential physiological functions.^26^ Vitamin D interventions effectively corrected hypophosphatemia, supporting their therapeutic potential for hypophosphatemic disorders. This finding is corroborated by clinical studies demonstrating the efficacy of vitamin D_3_, α-calcidol, and calcitriol in MBDP management.^1^^,^^27^^,^^28^ The 0Ca group in this model of early nutritional challenge exhibited the expected physiological adaptations to hypocalcemia, including elevated 25OHD, 1,25(OH)_2_D, and iPTH levels, accompanied by increased bone resorption markers and suppressed calcitonin levels.^29^^,^^30^ The strong correlation between 25OHD and 1,25(OH)₂D underscores that the bioactive form 1,25(OH)₂D is synthesized from its precursor and subsequently mediates its physiological effects.^31^ Notably, 0Ca animals did not develop hyperphosphatemia, likely due to 1,25(OH)_2_D-mediated FGF23 upregulation, which suppresses renal phosphate reabsorption. Although FGF23 levels in the 0P and 1/2P groups did not differ significantly from the NP group, the positive phosphate-FGF23 correlation (*r* = 0.396, *p* = .003) highlights FGF23’s role as a phosphate counter-regulator.^32^ This experimental evidence aligns with clinical observations that: (1) early postnatal phosphate deficiency suppresses FGF23 production and (2) persistently low FGF23 levels at 3-4 wk of age may serve as a predictive biomarker for subclinical hypophosphatemia in preterm infants.^33^

Our bone turnover marker analysis provided important insights into disease progression. While elevated alkaline phosphatase (ALP) is a well-established diagnostic marker for MBDP,^34^^,^^35^ our 0P group developed rachitic changes without significant BALP elevation, suggesting progression to a chronic deprivation state with osteoblast inhibition—a finding with direct clinical implications: early ALP elevation in preterm infants should prompt immediate phosphate status evaluation to prevent MBDP progression.^34^^,^^35^ This contrasts with reports of phosphate-BALP negative correlation in early disease stages,^13^^,^^36^ highlighting the dynamic nature of phosphate-dependent bone remodeling. Beyond phosphate homeostasis, our biomarker analysis delineated phase-coupled bone remodeling dynamics: (1) in the formation cascade, BALP (early-phase) strongly correlated with osteocalcin (late-phase), while PICP (mid-phase) exhibited inverse relationships with both BALP and osteocalcin, suggesting coordinated utilization of collagen precursors during osteoblast differentiation; and (2) the resorption cascade demonstrated parallel coupling, with TRACP (early-phase) positively associated with CTX-I release (late-phase), while calcitonin showed negative correlations with both resorption markers.^37^ Furthermore, vitamin D metabolites exerted complex bidirectional regulation of bone metabolism,^38^^,^^39^ as both vitamin D_3_ and calcitriol significantly elevated bone formation markers (*p* < .001), while their serum levels showed consistent positive associations with both formation and resorption markers. This bidirectional regulation aligns with reported dual actions of 1,25(OH)_2_D: stimulating osteoblastic collagen synthesis^31^^,^^40^ while simultaneously enhancing osteoclastic activity.^20–23^

When instituted in the neonatal period, phosphate deprivation caused significant HZ expansion and growth plate widening, whereas calcium deprivation resulted in marked reductions in these parameters. Most strikingly, phosphate deprivation combined with vitamin D metabolite administration induced pronounced growth plate atrophy, representing the most severe morphological alteration observed. Imaging studies revealed distinct mineralization pathologies: phosphate deprivation induced classic rickets with growth plate widening, trabecular thinning, and biomechanical compromise; calcium deficiency produced an osteoporosis-like phenotype with preserved tissue volume but reduced mineralization density. These findings corroborate previous reports that phosphate exerts 3-6 times greater influence than calcium on skeletal mineralization.^15^

Our experimental data demonstrated that while physiological vitamin D supplementation in the 0P group yielded modest skeletal benefits compared to complete deficiency in the 0P/D group, neither supraphysiological-dose vitamin D_3_ nor calcitriol rescued underlying mineralization defects despite normalizing sP. These findings reflect the well-documented yet complex dual nature of vitamin D’s skeletal actions,^20^^,^^31^^,^^40–42^ a phenomenon that mirrors current clinical debates about MBDP therapy. While some studies support high-dose vitamin D or active metabolites (eg, calcitriol) for improving phosphate homeostasis and bone health,^1^^,^^43^^,^^44^ others question their necessity and safety.^4^^,^^45^ Given these conflicting data, we emphasize that prolonged vitamin D or calcitriol therapy requires rigorous monitoring of 25OHD, iPTH, urinary calcium, and serum mineral levels to mitigate risks of nephrocalcinosis or hyperparathyroidism.^46^^,^^47^

Our study has several limitations inherent to the animal model and experimental design. First, while our neonatal rat model successfully recapitulates key features of MBDP, the use of discrete dietary phosphorus levels (0P, 1/2P, and NP) rather than a continuous gradient prevents precise quantification of dose-response relationships or the identification of specific threshold levels for impaired mineralization. Second, although we tested a range of vitamin D states, our model did not permit direct comparisons between different vitamin D analogs (eg, cholecalciferol vs alfacalcidol vs calcitriol) at clinically relevant doses, nor did it include comprehensive safety assessments, such as 24-h urinary calcium profiling or detailed renal histopathology—evaluations critical for translating findings into clinical practice. Finally, the fixed 6-8 wk endpoint may have missed critical transitional metabolic adaptations; a longitudinal design with serial monitoring of hormonal profiles, bone turnover markers, and in vivo imaging would provide a more dynamic understanding of the disease progression. These limitations, however, map directly to clear future research directions: establishing detailed phosphate dose-response curves, conducting head-to-head comparisons of vitamin D analogs, and implementing longitudinal protocols for metabolic phenotyping.

## Conclusions

This study establishes phosphate as the pivotal regulator of skeletal development in neonatal rats. Neonatal-onset phosphate deprivation causes severe rachitic changes and growth impairment, whereas neonatal-onset calcium deficiency leads to a distinct osteoporosis-like phenotype. Supraphysiological vitamin D cannot compensate for phosphate lack despite normalizing serum levels. Our work underscores that MBDP management must prioritize phosphate status and suggests that vitamin D monotherapy without phosphate supplementation is ineffective and potentially detrimental.

## Supplementary Material

Supplementary_Figure_S1_ziag007

Supplementary_Figure_S2_ziag007

Supplementary_Figure_S3_ziag007

Supplementary_Figures_captions_ziag007

## Data Availability

The datasets generated and analyzed during the current study are available in the Figshare repository under the embargoed DOI https://doi.org/10.6084/m9.figshare.30076273.v1. The data are embargoed until September 11, 2027, to allow for the publication of the findings. This version (V1) contains the primary data and codebook following the re-analysis with a length-normalized micro-CT ROI (5% of total tibial length) as requested during revision. During the embargo period, the data are available for peer review via the following private link: https://figshare.com/s/6ea78fe80c1752439978.

## Permission to reproduce material

Permission to reproduce material from other sources is not applicable.

## Clinical trial registration

Clinical trial registration is not applicable.
