# Supplementary figures and images for: Effects of graded phosphate deficiency and vitamin D intervention on growth, bone metabolism, and mineralization in a rat model of neonatal-onset metabolic bone disease
Source: JBMR Plus. 2026 Jan 16;10(3):ziag007. doi: 10.1093/jbmrpl/ziag007 (PMC12861084; doi:10.1093/jbmrpl/ziag007)

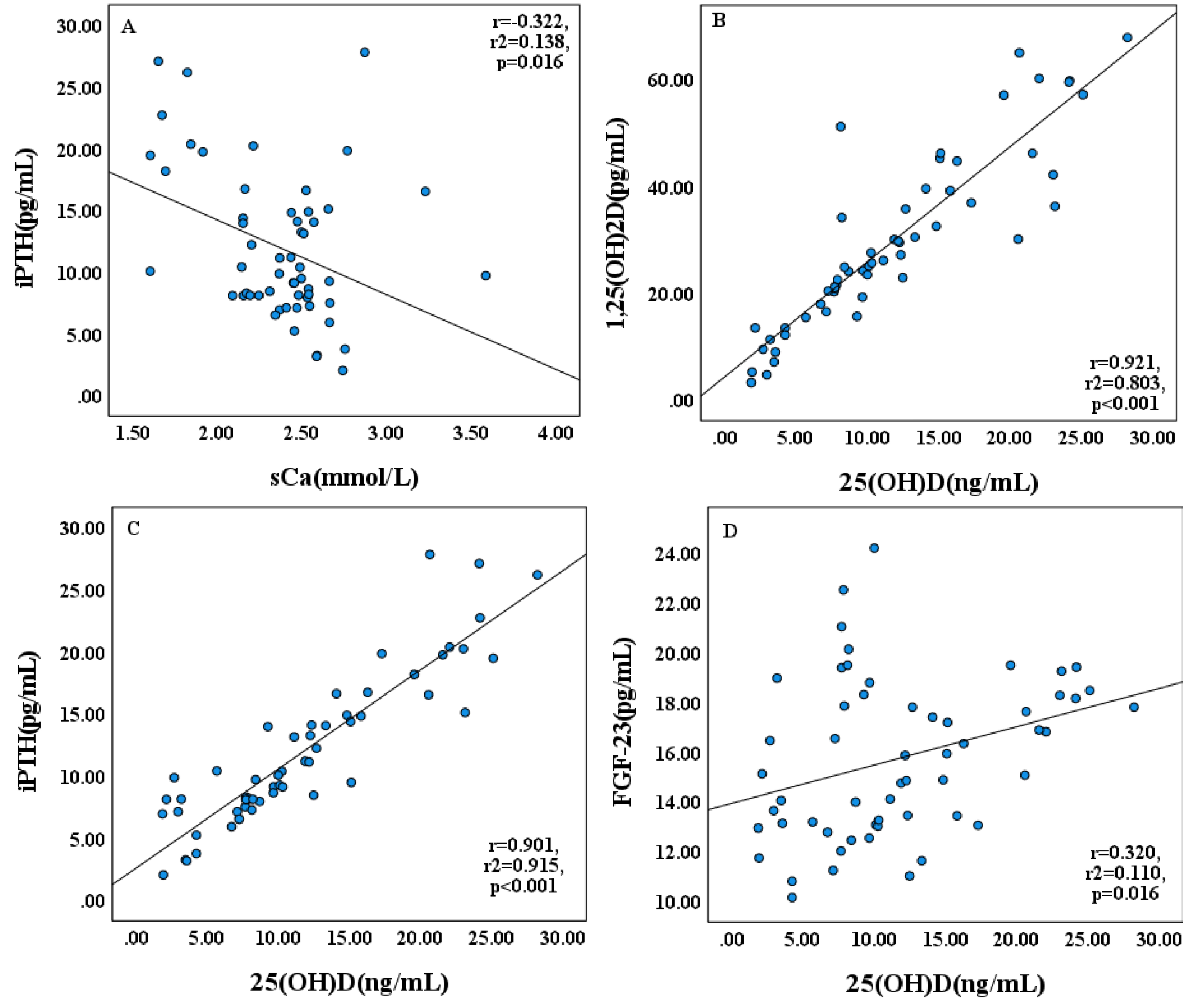

Supplement: Supplementary_Figure_S1_ziag007 [file supplementary_figure_s1_ziag007.jpeg]

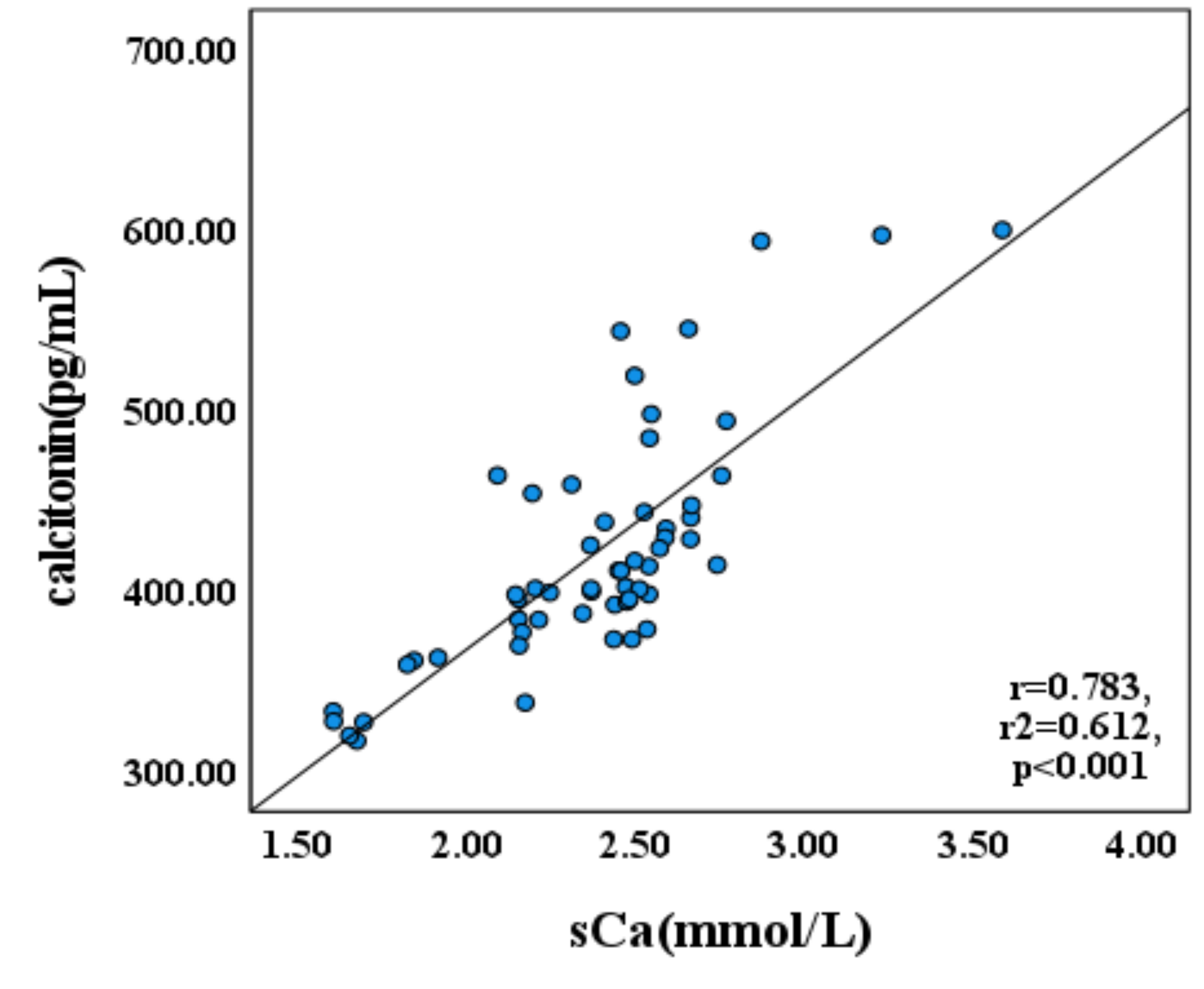

Supplement: Supplementary_Figure_S2_ziag007 [file supplementary_figure_s2_ziag007.jpeg]

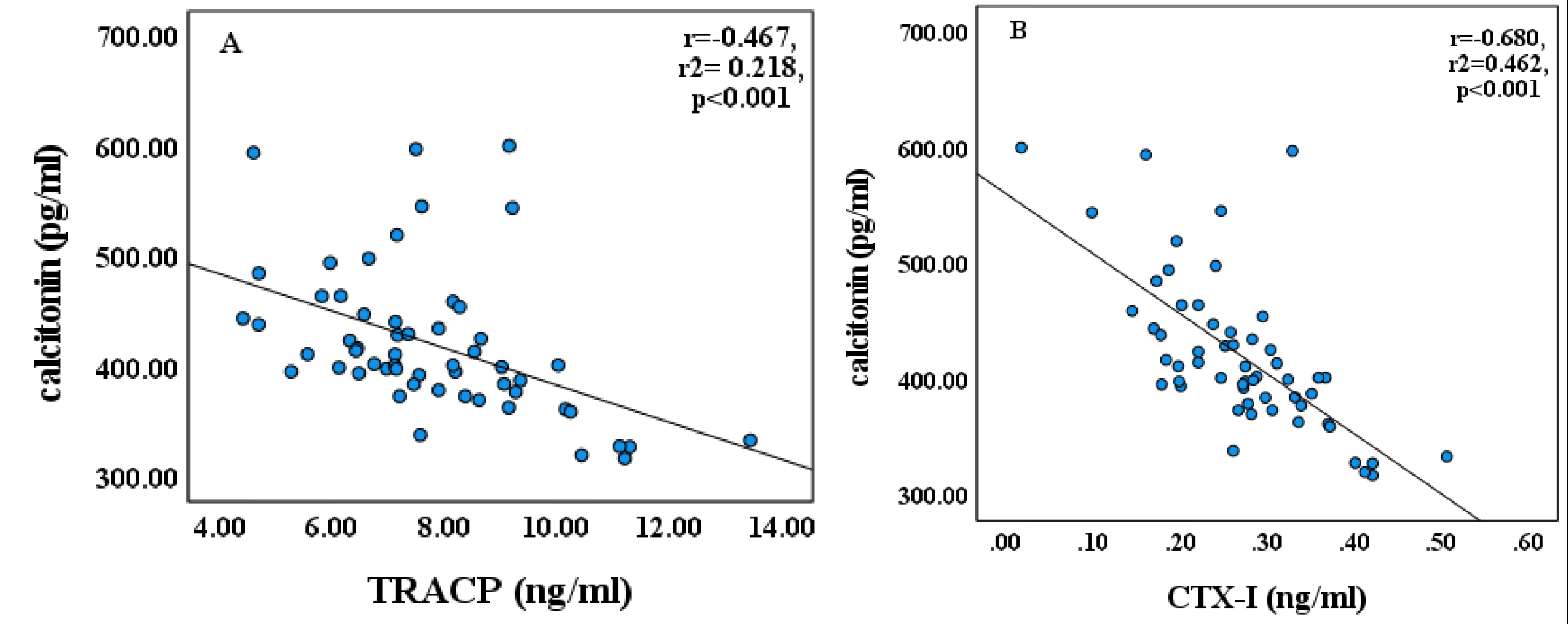

Supplement: Supplementary_Figure_S3_ziag007 [file supplementary_figure_s3_ziag007.jpeg]
