# Supplementary material for: Effects of graded phosphate deficiency and vitamin D intervention on growth, bone metabolism, and mineralization in a rat model of neonatal-onset metabolic bone disease
Source: JBMR Plus. 2026 Jan 16;10(3):ziag007. doi: 10.1093/jbmrpl/ziag007 (PMC12861084; doi:10.1093/jbmrpl/ziag007)
Supplement: Supplementary_Figures_captions_ziag007 [file supplementary_figures_captions_ziag007.docx]

Supplementary Figure S1 Correlations between serum calcium and phosphotropic hormones.

Linear regression analyses of serum parameters in neonatal rats under dietary interventions: (A) sCa vs. iPTH; (B) 25(OH)D vs. 1,25(OH)_2_D; (C) 25(OH)D vs.iPTH; (D) 25(OH)D vs. FGF23. Data from all experimental groups were pooled for analysis (n = 56). Solid lines indicate the line of best fit. Abbreviations: sCa, serum calcium; iPTH, serum intact parathyroid hormone; 25(OH)D, serum 25-hydroxyvitamin D; 1,25(OH)_2_D, 1,25-dihydroxyvitamin D; FGF23, serum fibroblast growth factor-23.

Supplementary Figure S2 Correlation between serum calcium and calcitonin.
Linear regression analyses of serum calcium and calcitonin: sCa vs. calcitonin. Data from all experimental groups were pooled for analysis (n = 56). Solid lines indicate the line of best fit. Abbreviations: sCa, serum calcium.

Supplementary Figure S3 Correlations between calcitonin and bone resorption markers.

Linear regression analyses of calcitonin and bone resorption markers: (A) TRACP vs. calcitonin; (B) CTX-1 vs. calcitonin. Data from all experimental groups were pooled for analysis (n = 56). Solid lines indicate the line of best fit. Abbreviations: TRACP, tartrate-resistant acid phosphatase; CTX-I, C-terminal telopeptide of type I collagen.
